# Cybernetic Principles in Psychophysiology: Their Significance and Conclusions for Palliative Care

**DOI:** 10.3390/healthcare12151510

**Published:** 2024-07-30

**Authors:** Michael Brinkers, Giselher Pfau, Beatrice Thielmann, Irina Böckelmann

**Affiliations:** 1 Pain Outpatient Clinic of the Department of Anesthesiology and Intensive Care, Faculty of Medicine, Otto von Guericke University Magdeburg, Leipziger Str. 44, 39120 Magdeburg, Germany; michael.brinkers@med.ovgu.de (M.B.); giselher.pfau@med.ovgu.de (G.P.); 2 Institute of Occupational Medicine, Faculty of Medicine, Otto von Guericke University Magdeburg, Leipziger Str. 44, 39120 Magdeburg, Germany

**Keywords:** interoception, personality traits, tipping point, subjectivity, identity in transition

## Abstract

Palliative care is dedicated to terminally ill patients with advanced disease, regardless of diagnosis, under the overarching premise of optimizing quality of life. This narrative review examines the extent to which principles of cybernetics and psychophysiology underlie this approach. Psychophysiology researches the physiological equivalents of psychological states and traits such as activation and individual reactivity, the interoception and the personal characteristics. Cybernetics specifies these principles, which are possible by understanding terms such as “psychophysiology” or “cybernetics” or “self-organization/autopoiesis”. The meaning of these terms for palliative care can also be elucidated in relation to the terms “biofeedback”, “consciousness”, “pain”, and “anxiety”. The common themes of cybernetics and psychophysiology are environment, subjectivity, personality characteristics, the difference between time scale separation in cybernetic systems, and real-time procedures in environment and rhythm. These lead to special therapies based on psychophysiology, such as consciousness training. The concepts of quality of life, causality, the biopsychosocial model, therapy, and autonomy are examined as palliative care concepts. The equivalents can be described from the perspective of cybernetics. For some palliative care-related terms, cybernetic thinking is already present (quality of life, autonomy, symptom control), while for others, it is not (biopsychosocial). Cybernetic terms (complexity, stability, identity, rhythm) are still used to a lesser extent in palliative care. Terms like genetic basis are common in cybernetics and psychophysiology to explain the identity of the subject in transition. Identity, on the other hand, is the basis of the concept of dignity in palliative care. Psychophysiology investigates disturbances like pain and psychological illnesses, which are also present in palliative care. Psychophysiology, cybernetics, and palliative care have subjectivity and resources in common. Therapies based on cybernetic principles of psychophysiology can also be used for symptom control in palliative care in the oncology setting.

## 1. Introduction

The aim of this narrative review is to analyze the medical field of palliative care in terms of its underlying cybernetic principles. However, as the review by Schmidgen [1] shows, cybernetics is a broad field that also includes the psychology of perception.

Psychophysiology researches and describes the relationships between psychological processes and physiological principles. This opens up a wide field from behavior, emotions, and mental disorders to brain function, transmitter balance, and physiological parameters such as respiratory rate and heart rate [2]. The border area between “normal” and the onset of a disease process was particularly important for this review. Psychophysiology is based on three points [2]: 1. Physiological processes are measurable (EEG, 24 h ECG); 2. They are not identical in all people, but individual; and 3. There is no compelling connection between observable/observable physiological parameters and the conditions perceived by the patient.

In this review, for example, this applies to pain and anxiety, which are the most frequently reported symptoms in palliative care patients [3]. The psychophysical make-up of the individual and the significance of environmental conditions are also topics of cybernetics. This review begins with the following facts of cybernetics:

Medical history: Each system has its own “history” [4]. In biological systems, this results in a post-correction state that is different from the pre-correction state. For example, after the body’s defense system fights off a new pathogen, it is not exactly the same as it was before the defense (e.g., due to the formation of new memory cells), or repeated confrontation with a stimulus or stressor results in habituation to it, i.e., a physiological adaptation or habituation response.

Identity in transition: It can be defined as the identity of a subject that is in contact with a constantly changing environment [5]. This view is the basis of this article because it is this approach to medicine that has enormous importance in understanding the patient’s illness. The conclusion is as follows: LEARNING BY ADAPTATION is preferable to LEARNING BY REPEAT. These reflections already culminate in the question posed by von Uexküll and Wesiak [4]: “Where can we anchor the identity of systems that constantly are changing their permanence and their structure?” Their answer was the following: “This paradox is a characteristic of all living entities” [4].

Hierarchy: The error in linear–causal thinking is the assumption of hierarchy. According to Weiner [6], this does not necessarily have to be the case because, for example, mental processes do not require command cells [6].

### 1.1. Cybernetic Systems Offer Three Advantages

Cybernetic systems have three advantages [5]. First, according to systems theory, interactions with the (social) environment are implicit in cybernetic systems. For example, the “thyroid gland” system also depends on food intake from the environment. Thus, the social factor does not need to be taken into account. Second, the system changes slowly by constantly trying to adapt. For example, a high heart rate variability (HRV) is a sign of good regulation of the cardiovascular system in terms of good adaptability [7]. HRV can also be used as an indicator of the psychophysical state of the organism and the limitation of adaptability to biopsychosocial problems [8]. Activation of regulatory mechanisms controlling cardiovascular homeostasis is associated with the functional state of the organism [7]. Third, the activation of the sympathetic or parasympathetic nervous system allows us to assess the adaptability of the whole organism to a particular situation or stimulus, such as pain [9]. This can explain why a chronic symptom (e.g., pain) may not occur despite repeated stimuli.

### 1.2. The Role of Psychophysiology

The commonality of cybernetics and psychophysiology is the individual and subjective state of the patient. Cybernetics is a theoretical model. Psychophysiology is the study of the processes of diseases such as pain or anxiety [2]. Therapies are derived from this. The advantage of cybernetic principles in psychophysiology is that they emphasize subjectivity in their importance for optimizing personal interoception through therapies like music [10], aromatherapy [11], or biofeedback [12].

The keywords mentioned here have not yet been included in books on palliative care [13]. This review therefore attempts to investigate which concepts of palliative care already have a cybernetic basis and which concepts of palliative care would still need to be included if they were to turn to the principles of cybernetics (and psychophysiology). 

We also choose palliative care as an example of psychophysiological methods because palliative care has the most subjective component.

The first aim of this paper is to outline the relevance of system theory/cybernetic considerations in their psychophysiological equivalents for palliative care. The second aim is to work out what similarities arise in detail from cybernetics and psychophysiology for palliative care. These considerations are based on the findings of the narrative review.

We are trying to answer the following question: “What can psychophysiology and cybernetics offer for therapies based on cybernetic principles in palliative care in the oncology setting?

## 2. Materials and Methods

A literature search was conducted to show how numerous the papers on cybernetics and psychophysiology are, while papers on these topics in the context of palliative care are rare.

Initially, the intersection of cybernetics, psychophysiology, and palliative care was searched under the combination of the terms with the term “cybernetics”.

For cybernetic principles in palliative care, the literature was searched for the terms “system theory”, “autopoiesis”, and “bio-psycho-social” in combination with the term “palliative care”.

In addition, the terms “psychophysiology” and “palliative care” were combined with the terms ”anxiety”, “pain”, “self-organization”. In addition, the terms “biofeedback”, “hypnosis” in combination with “palliative care” were entered to investigate the benefits of psychophysiological methods in palliative care.

**Figure 1:**
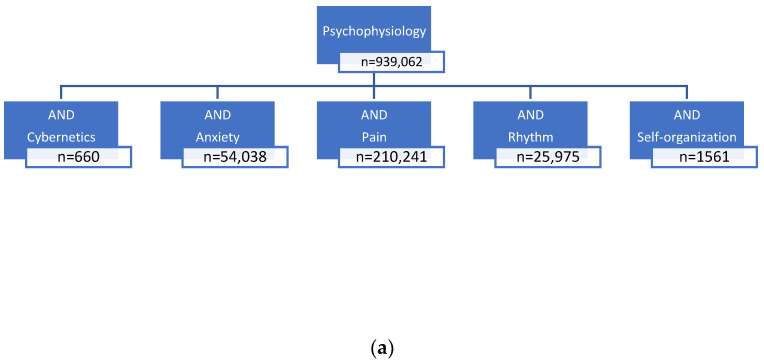

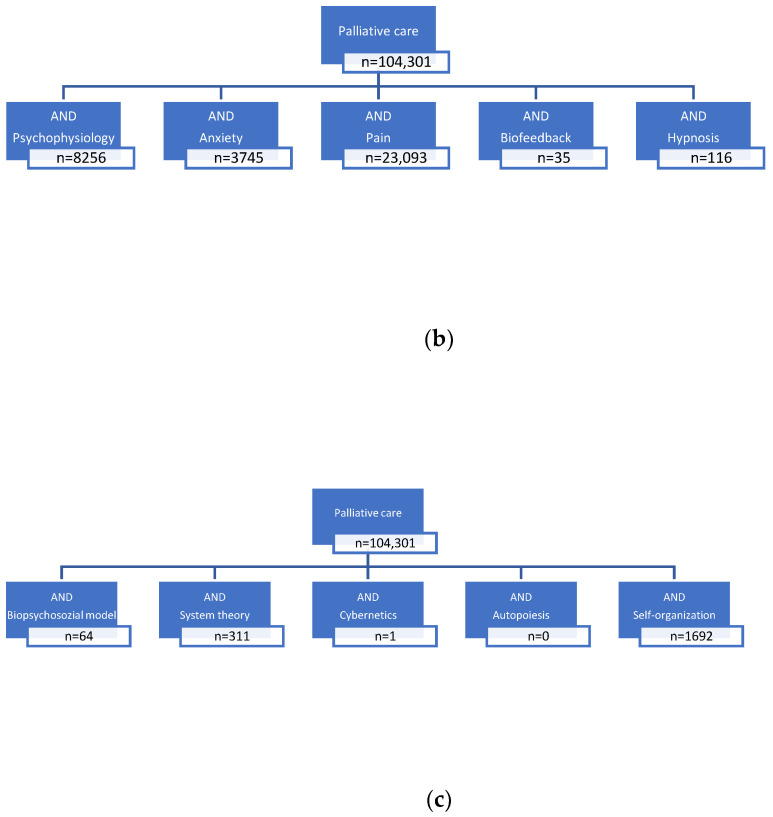
(**a**) Results of literature search of psychophysiology. (**b**) Results of literature search of palliative care, part 1. (**c**) Continuation of results of literature search of palliative care, part 2.

We used PubMed considering articles in the German and English languages published until 12/2023 (Figure 1a–c). This time frame was chosen because we have not yet found a comparable review and therefore wanted to include all work on this question up to December 2023. Each search included the above search terms. All papers (meta-analyses, systematic reviews, cohort studies, etc.) were considered first.

Inclusion criteria: Published articles were considered if they were written in English or German and were published up to the end of 2023. 

Exclusion criteria: other languages, no access to the abstract or to the full article. 

The results were compared with those of a literature search using the term “biopsychosocial model”. In addition, textbooks on palliative care as well as graduate and doctoral theses on the subject available on the Internet were consulted. 

## 3. Results

The literature search (Figure 1a) revealed a large number of articles on pain in psychophysiology (*n* = 210,241), anxiety in psychophysiology (*n* = 54,038), and rhythm in psychophysiology (*n* = 25,975). Articles on cybernetics in psychophysiology are rare (*n* = 660). The article search revealed a large number of studies on pain in palliative care (Figure 1b). Articles on biofeedback are extremely rare (*n* = 35). Articles on autopoiesis in palliative care are completely missing, and very few describe cybernetic approaches (Figure 1c). The terms “autopoiesis” and “cybernetics” are poorly represented in the context of palliative care. There is no research on the combined terms of cybernetics and psychophysiology in the palliative care literature.

Therefore, an attempt was made to find equivalents for the most important terms in the palliative care literature (quality of life, personal dignity/autonomy, symptom control, biopsychosocial model, causality). Starting from this point, the search for literature from cybernetics and psychophysiology via the specialized literature yielded 111 sources.

### 3.1. Cybernetics Principles in Psychophysiology

The field of psychophysiology examines the relationship between mental states (states and traits) and their possible accompanying physiological states/bases. For the present review, interoception and personality traits are of particular importance in psychophysiology [14]. These are based on cybernetic principles.

#### 3.1.1. Cybernetic Principles of Psychophysiological Terms

A common term of cybernetics and psychophysiology is subjectivity. It has three aspects.

Interoception refers to the subjectivity of the perception of one’s own psychophysical states. The perception is influenced by the psychosocial situation of the individual as well as the causes of their psychophysical states assumed by the individual.

The psychosocial situation (environment), in turn, is more precisely captured by the term “bio-psycho-social”. In a recent publication, Brinkers pointed out the weaknesses of this concept, including the lack of a definition of the relationships between the three components [15]. When Popkirov therefore speaks of a bio-psychosocial model that does not assume causal relationships, this is already an extension of the cybernetic idea of a circular model [16]. Doering and Söllner therefore also speak of a cybernetic biopsychosocial model [17].

Subjective perception was also identified by Brinkers as a key factor in assessing the overall clinical picture [15]. This is consistent with the everyday experience that the intensity of pain with the same underlying organic condition (e.g., cancer) is reported differently by each patient. It also corresponds to Vogelzang’s findings that conditions such as pain and fatigue are weighted differently by patients than by physicians in terms of their personal significance for the respective patient [18,19]. In cybernetics, subjective perception is based on the autonomy of the patient [20].

Personality traits: Brinkers recently pointed out the importance of disposition for the development of physical and/or psychological complaints. In psychiatry, this corresponds to the diathesis–stress model [15].

In addition to the innate characteristics, the acquired characteristics of an individual are added over the years, as described in the Meikirch model [15]. According to this model, disease is a mismatch between the individual’s potential and the demands placed on it.

In psychophysiology, this corresponds to system overload [21]; in cybernetics, it corresponds to the tipping point at which counter-regulation of a disruptive factor becomes positive reinforcement. What all three have in common is that they are subjective processes that do not correspond/do not have to correspond 1:1 to objectifiable/observable states.

#### 3.1.2. Further Common Themes of Psychophysiology and Cybernetics

Time course or the relevance of real time: While psychophysiology with its measurement methods is concerned with measuring changes that occur in real time, cybernetics says that complex processes have time delays. For example, information about interactions between systems is not passed on immediately, but with a delay. A complex system adapts to constant changes in its environment, decouples itself through its reaction time, i.e., the delayed adaptation, especially with increasing size, and at the same time gains stability itself [22].

This delay also enables the individual to develop an idea of what has changed [22]. Cybernetics calls this the separation of time scales.

Rhythm: Many physiological processes in the body are characterized by a rhythm, such as menstruation or sleep. Sleep disorders in particular show the dependence of the body’s own rhythmic system on various environmental factors such as exposure to light and darkness, genetic factors, gender, or developmental age [23]. Therapies such as music against anxiety and pain are explained by authors by the fact that the rhythm of the selected pieces is subjectively perceived as appropriate by the patient [10].

#### 3.1.3. Circulatory Model

Biofeedback used in psychophysiology based on the circulatory model [24]. The assumption here is that disruptive factors have led to a dysregulation of a physiological system. The cybernetic principle is counter-regulation instead of eliminating the disruptive factors. The positive adaptation processes become effective when one’s own skills and abilities are improved. Negative effects occur when so-called continuous performance limits [21] are exceeded.

The aim is for the practitioner to regain control over physiological mechanisms such as heart rate, breathing rate, or blood pressure. This leads to possible areas of application such as headaches and hypertension. The dysregulation model also includes the fact that biomedical therapies themselves can lead to dysregulation and thus impair the natural ability to self-regulate [24].

### 3.2. Cybernetics of Living Systems (Circulatory Model)

According to Vossius (1980), cybernetic systems (the circulatory model) have three characteristics: (1) the dualism environment vs. personal factors, (2) the negative feedback controller (counter-regulation), and (3) the variable setpoint [5]. The cybernetic system is described below.

Dualism: The first characteristic is the perception of the patient’s organic condition as the result of a mental process. This is influenced by the person’s general physical condition, which in turn is dependent on external social conditions, i.e., personality traits and environment. For example, the perception of pain in the case of severe physical changes (e.g., tumor) is different from the perception of pain in the case of a mental disorder [25]. There is a direct interaction between each person and their environment: the reception of information by the receptors and the influence on the environment by the effectors. Thus, it is not a hierarchy of systems (biological, psychological, environmental) but a description of the two-way ongoing influence of systems working in parallel, e.g., pain, cancer, and mental disorders [26].

**Figure 2:**
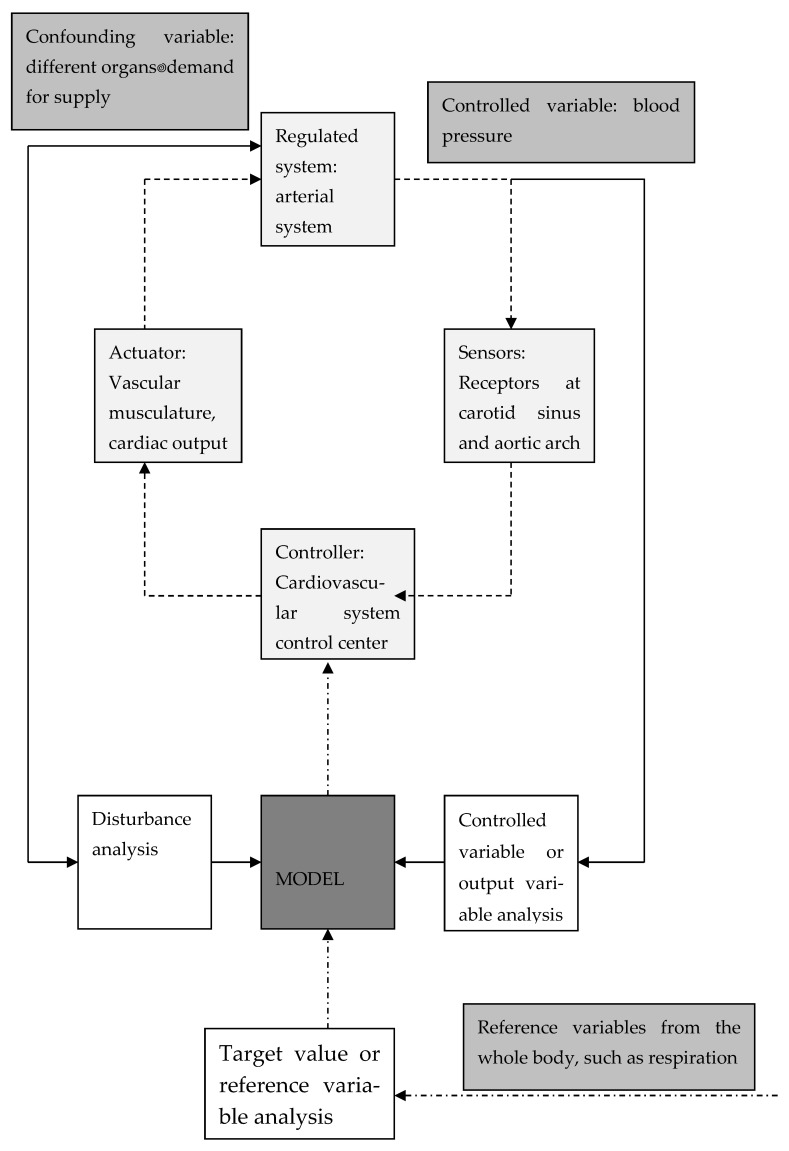
Cybernetic system exemplified by arterial blood pressure regulation (adapted from Vossius [5]).

Controller: The second feature of the cybernetic model is counter-regulation. A part of the circulatory model, the controller, is not the apparatus that automatically counteracts but a central instance that detects the deviation of the previously measured actual value from the offered target value. It releases the control signal, which then regulates an adequate energy flow (coping mechanisms) in the peripheral actuator. For example, in studies of the cardiovascular system, Vossius [5] regards the cardiovascular center in the CNS as the instance that regulates the arterial blood pressure and the peripheral vessels and the heart as the coping mechanism (Figure 2). The system constantly synchronizes the actual value with the target value.

**Figure 3:**
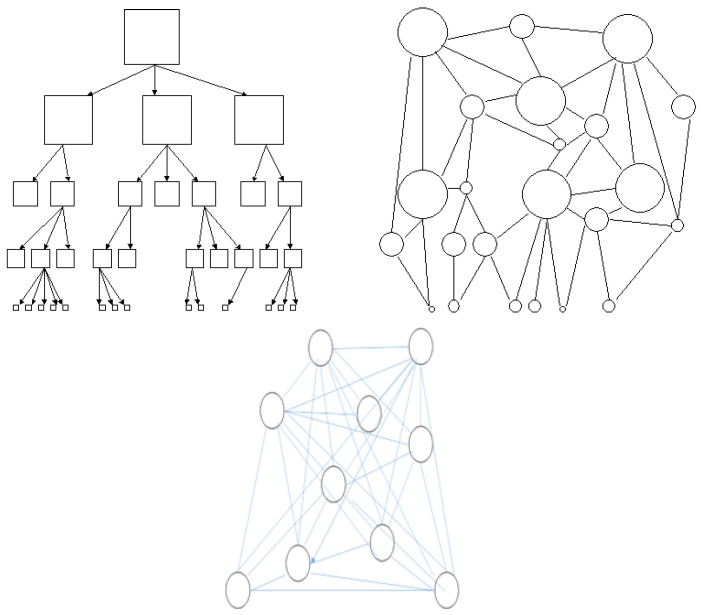
Comparison of hierarchy (example on the left: military) against the complexity of the self-organizing network (example on the right: e.g., brain, human system) (due to Wolf [31]: the larger the squares or circles, the more important. In the lower figure, significant structural units are decentralized. Networking alone is not a stability characteristic. Completely networked systems are unstable (Vester [30]).

Target value: The third feature, the target value of the system, is not unique but depends on the requirements of the overall system. This term implies three further conditions:

1. Cybernetic systems are complex: Systems are complex when their parts are interconnected by mutual, permanently changing relationships. The parts of the system themselves can also undergo changes at any time.

2. Autopoiesis and environment: The process that enables change is autopoiesis, or self-organization [27,28]. Autopoietic systems do not receive their structures ready-made from the environment, but must construct them through their own processes. The output of the elements of a system always produces the exact system that produced those elements. Autopoiesis has a bidirectional approach: (a) the network system and (b) the closed system. The network system refers to the network-like characteristics of cybernetic systems. Autopoietic processes need not to be part of the system hierarchy depicted in Engel’s model [29]. For example, impulses that require the emergence of processes in cardiovascular regulation need not originate from pacemaker cells at the top of a hierarchy in the cortex but originate from networks/circuits (Figure 3) [30,31]. As a closed system, elements do not enter the (circular) process from the outside [32]. The dualism of “system-environment difference” [32], interdependence, and autopoiesis are important concepts when studying interaction with the environment. In this context, any element outside the observed system is to be considered as environment. Rather, these elements provide a stimulus for the system in question to change, but they are not/will not become part of the system. For example, the body’s immune systems normally produce B and T cells in response to external stimulation; these cells are not imported into the body from the outside. Cell production is a process that is always the same. Therefore, complex systems are “operationally closed” and “autonomous” [33,34]. This is the precondition for answering two questions: (1) Which resources does the system need for self-organization, and (2) which events in the environment (e.g., pain stimuli as a disturbance factor, see below) are perceived as stimuli, neutralized or potentiated? [35].

3. Changes in autopoiesis: Many control systems have a fixed target value that is set repeatedly, such as the pH of the stomach. The controller in cybernetic systems does not have a fixed value to regulate against, but a target value that depends on the whole organism. Furthermore, there are two time aspects: decay/temporalization and preformation. Schiepek (1990) mentions a temporalization of the system components. In biological cells, for example, the corresponding molecular components are synthesized from low-molecular-weight components, but are broken down and eliminated through the cell membrane (e.g., opiate receptors). The non-material substrate remains, but the ordering principle of the process is “creating receptors”. The same is true for to psychological and social processes [35]. The other aspect is preformation. Living systems use predictive models for future changes in systems and environments [36]. This includes conditioning and learning processes, such as the memory cells of the immune defense or the genome [37]. According to Luhmann (1997), all processes therefore always have a dual function [38]. Processes determine the historical state of the system, which is the basis for this system to perform the next processes, i.e., they determine the system as given in such a way and not otherwise, and all processes create structures in the form of selection schemes that enable recognition and repetition. From this continuity arises the identity that autopoietic systems keep their organizations (networks) invariant through all their production processes, regardless of the interactions.

## 4. Discussion

The aim of this work is not to present the field of cybernetics together with psychophysiology in a comprehensive manner in order to show the significance of cybernetics and psychophysiology using the example of palliative care.

Rather, after reviewing the literature, the approaches, principles, and goals of palliative care are formulated and compared with their cybernetic equivalents and psychophysiological principles. This will be discussed in three sections.

Section I discusses the overlap of cybernetic principles and psychophysiology. What are the terms used in palliative care?

Section II discusses the equivalents of cybernetic principles in palliative care. Which terms of palliative care can be traced back to cybernetic principles? Which cybernetic principles are not yet anchored in palliative care?

Section III discusses the importance, significance, and conclusion of “cybernetic” psychophysiology for palliative care. What is the role of the subjectivity of psychophysiology and therapies based on it in palliative care?

### 4.1. Cybernetic Principles in Psychophysiology

Psychophysiology deals with the possible relationship between emotions, consciousness, and behavior on the one hand and physiological bases such as transmitter balance/hormones, brain activity [39], and motor skills on the other. This opens up a wide field of investigation possibilities, culminating in philosophical considerations such as mind–body dualism. In this review, only some aspects of psychophysiology can be highlighted as having a possible intersection with cybernetics.

#### 4.1.1. The Subjectivity

Against the background of subjectivity (in cybernetics and psychophysiology), these are the environmental conditions, the subjective perception of psychophysical states [1], the innate and acquired potentials/dispositions [14], and the personality traits. In addition, there is a preoccupation with the uniqueness of the individual in his or her individual reactivity.

In cybernetics, the respective state of an individual at a certain point in time is examined. This is initially depending on several components such as (a) the basics, (b) the mechanism of a cybernetic model (counter-regulation), and (c) the influencing factors [5]. The basis in cybernetics is the circulatory model, the operational closedness with simultaneous energetic openness. The mechanism of the model is the counter-regulation of a disturbance factor [5], dealing with the possibility of the compensation of a disturbed state without a setpoint/target value. Influencing factors result from internal and external conditions as well as the psychosocial environment. 

##### Circulatory Model

Concrete thematic overlaps between psychophysiology and cybernetics therefore exist in the following points of the circulatory model:

Dualism: Both fields therefore have environmental conditions in common.

While this is an inherent necessity in cybernetics due to the energetic openness of the system, it is specifically formulated in psychophysiology. Reference is made to the bio-psycho-social model. Cybernetics assumes a difference between the observed system and its environment. This difference maintains the process of adapting the system to the environment [5].

Counter-regulation/Controller: When additional disruptive factors occur, the system itself attempts to compensate for the disruption without eliminating it [5]. This is performed by a controller, which in turn specifies target values, which are formed by a model based on the disturbance, the current state, and the required standard of the environment.

Target value: This is the transition to the subjectivity of psychophysiology: Individuals have the ability to compare their desired condition with their actual state [40]. The desired target value is therefore always relative and not fixed [5].

##### The Counter-Regulation: Cybernetics of Psychophysiology in Illnesses/Diseases

Dysregulation: From a cybernetic perspective, dysregulation can be the cause of counter-regulation or the consequence of unsuccessful counter-regulation. Therefore, the basis of the circulatory model with counter-regulation is particularly important as an explanation for chronification [41]. The point of counter-regulation reversal in the sense of dysregulation [24] is of particular importance in the development of illnesses/diseases.

Psychological and psychosomatic diseases are the subject of psychophysiology [14]. The present review focuses on the topics of consciousness, anxiety, and chronic pain, which are addressed by psychophysiology [42], but are also important in palliative care [11,43,44,45,46]. In psychophysiology, it is known that in phobics there is a close correlation between the extent of heart rate acceleration and the subjectively assessed fear. This is not only the case when confronted with the object, but also when the phobic object is imagined or when the phobic object is presented in a slide [42]. 

In psychophysiology, pain is considered an example of a complex process in which not only the simple transmission of information takes place, but also the processing of peripheral stimuli involving emotional and cognitive states, taking into account previous experiences and plastic processes such as reorganization [39].

Central instance: In humans, there is an extension of the structure of cybernetic circulatory models. According to Schaefer (1972), the control loop of human action possesses an actuator that can change the setpoint itself: the cerebrum [47]. The controller also has the task of compensating for differences. Now, however, it can achieve this task not only by changing the actual value but also by changing the setpoint.

Psychophysiology also speaks of a “central governor” that regulates activities to ensure that homeostasis is maintained and physical damage is avoided [14]. “The concept of dysregulation in human illness is used to elucidate how environmental factors can modulate the central nervous system and affect homeostatic, self-regulatory control of peripheral organs. When feedback from peripheral organs is disrupted, dysregulation occurs” [24]. This leads to physiological instability and functional disease/illnesses.

This is the basic assumption for psychosomatic illness [14], which in turn is the subject of psychophysiology.

The loss of control by the brain is also discussed in sudden infant death syndrome [48]: “Breathing is, by default, an autonomous function, but breath control is learned. If there is not a smooth transfer of function at the time when a successor system (one that enables autonomous breathing to be overridden by voluntary control) takes over, breathing may cease, without any overt cause being detectable, even with a thorough postmortem examination”.

#### 4.1.2. Psychophysiological Therapies of Illnesses/Diseases

Cybernetics initially represents a therapeutic model, the effects of which are always transferred to a defined area and adapted anew. To this end, further developments have been made in terms of content, allowing adaptation to special clinical pictures, such as muscle pain [25,49].

On the other hand, psychophysiology offers possible applications that help individuals to optimize their subjectively perceived state. Based on the cybernetic principles mentioned above (dualism, counter-regulation, controller, variable target value, time separation, rhythm), there are psychophysical therapy options.

The proposals initially follow the stress–strain concept by [21]. Stress is defined as all external environmental factors affecting a person, whereby the degree of stress always depends on the individual conditions [50]. In the temporal sequence of stress, a distinction is made between short-term stress reactions and the long-term consequences of stress, which themselves act as stressors and are involved in psychophysical disorders [21]. On the other hand, according to the Meikirch model, it is possible to compensate for the individual differences by giving the sick individual more resources for the requirements or by changing the requirements [15].

Compensation: This can be achieved through rhythmization/counter-regulation, such as through biofeedback [12,24,51] or hypnosis [3,52] or music [10].

Deceleration of the person: According to Kaiser, there are several ways to decelerate personally: (a) switching off from work, (b) relaxation (this is where biofeedback, massage, and aromatherapy come in), (c) self-determination of action (Kaiser mentions that an action is perceived as relaxing when both the time and the type of activity are chosen by the individual), and (d) experiencing challenging but not overwhelming activities. These are therefore usually perceived as relaxing because they allow a broadening of horizons and the development of skills.

According to Loew (2019), there is the possibility of “decelerated breathing”, which has the same roots as the SIDS problem described above. The central instance of the will takes control of the innate breathing reflex [53]. The feedback mechanism also works here. This can lead to a reduction in blood pressure, a slowing of the heart rate, and an economization of cardiac output [54]. According to Rösel and Burian (2023), an important method to reduce pain is for patients to consciously deal with their cognitions and emotions [55]. This can be carried out through so-called “Acceptance and Commitment Therapy (ACT)”. This not only creates resources, but also promotes psychological flexibility by encouraging (pain) acceptance.

Deceleration of the environment: This includes physical exercise [21]. It fulfills all three of the above-mentioned characteristics of recovery of mental switching off, relaxation, and self-determination. However, this only applies if the benefits justify the effort in the subjective perception [56]. This also includes fixed breaks [21].

According to Wolfe et al. (2023), the ability to decelerate is a key component of any successful rehabilitation program [57].

### 4.2. Cybernetics in Palliative Care

From the perspective and on the basis of the cybernetic principles mentioned above (dualism, counter-regulation, controller, variable target value, time separation, rhythm) it has to be discussed to what extent palliative care concepts such as quality of life have a cybernetic basis and to what extent cybernetics provides concepts that are not yet used in palliative care and do not form a basis for action.

The discussion is therefore divided into two parts:

Section 4.2.1 discusses palliative care concepts and their cybernetic equivalents;
Section 4.2.2 debates cybernetic terms without corresponding palliative medicine equivalents.

#### 4.2.1. Palliative Care Concepts and Their Cybernetic Equivalents

Palliative care concepts and their cybernetic equivalents include the viewpoints of quality of life, the causality and consequence model, the biopsychosocial model, therapy vs. symptom control, and patient autonomy.

Quality of life: The WHO has defined palliative care as “treatment of patients with incurable, progressive and advanced disease with limited life expectancy, for whom the main goal of care is quality of life”. From a palliative care perspective, this includes the following aspects. According to Husebø and Mathis (2017), quality of life means (a) quality instead of quantity and (b) answering the following question: what are the consequences of the treatment for the patients? [58]. According to Mounsey et al. (2018), quality of life means multidisciplinary treatment, and according to Latimer and Dawson (1993), interdisciplinary treatment [59,60]. According to Wille et al. (2021), interdisciplinarity is important in palliative care [61], which means that there are skills that go beyond one’s own profession (Center for Integrated Health Solutions). This indicates that in a multiprofessional team, individual team members contribute to a common pool of knowledge and skills.

From the perspective of cybernetics, first, the state of counter-regulation in cybernetic systems is not the maximum of the system but its optimum [5,30,31,41]. This aspect was formulated in the Yerkes–Dodson law [62]. However, when this (maximum) occurs, the entire system becomes susceptible to malfunction [5]. Second, in applied cybernetics, the following key points are relevant: the question of the consequences of treatment is one of several goals to be considered in therapy, new subjective equilibrium relative to the patient’s own abilities, data reduction by pattern recognition, alternative possibilities in therapy, and creating a more disturbance-insensitive system (increase responsiveness) [41]. Third, interdisciplinary implies an absence of data accumulation by many specialists; instead, pattern recognition and the associated data are reduced to essential key components and the networking of these components [30].

Causality-and-consequence model: In classical medicine, the patient’s current condition is often attributed to one disease, e.g., lung cancer caused by smoking [63]. In parallel, however, there are other models, such as the multifactor model for schizophrenia [64].

From the perspective of palliative care, causality of symptoms and of therapy means non-curative treatment [65]. In this context, palliative care takes the path of a complex of causes [63] over the “total pain” concept [66]. What the total pain concept lacks is the relativity of factors to each other. It is not a single confounding factor that always produces the same effect, but the symptom is the overall result of other factors that may mitigate or amplify the single confounding factor, defined as the diathesis–stress model [64]. Curative therapy, the treatment of the “cause”, is possible in palliative care, but only if the benefit is greater than the risk for the patient [67]. Otherwise, the main role of palliative care is not curative therapy but symptom control [67].

From the perspective of cybernetics, the symptom is already the endpoint of a process [5]. The cause of the suffering cannot be derived proportionally from the causative organic factor. Conversely, the present state of the patient is more than the sum of the determined organic changes [30]. Mechanical systems have a direct cause–effect relationship: a certain cause leads to a certain result. System-theoretical cybernetic considerations are different: there is a continuous development. Therefore, there is not one triggering/causing factor in a cause-effect relationship, as suggested by classic stress research papers or modern flowcharts [68]. There is no certainty about the appearance of the final state [5,69]. The consequence is uncertainty and fuzziness instead of exactness [30].

This consequence is based on two concepts, nonlinearity and the fuzziness of biological systems.

According to Vossius (1980), nonlinearity must be emphasized in biological systems. Conclusions from physical systems are not entirely transferable to biological systems [5]. Physical systems are predictable, whereas biological systems are not because they are nonlinear. Table 1 shows a comparison of physical and biological systems.

Weiner’s (1994) concept of nonlinearity concludes that (a) the shape of biological systems cannot be predicted on the basis of their individual elements, (b) a small change leads to disproportionate changes, and (c) the factor time becomes a critical variable (see above problems [6]. According to Glaser’s (1997) information theory, the flow of certain information is not 100% predictable and cannot be transferred from one autopoietic system to another [69].

The second property, fuzziness, means that biological systems cannot design the order in the system in detail. This is only possible in abstract features through the fundamental rules [6]. Also, the behavior of a system also cannot be captured from the properties of its individual components [30]. For example, oxygen and hydrogen do not explain the properties of water. This means that their mode of process is different because of certain features. They do not give a response that is proportional to the input signal (system stimulus) [5]. Therefore, the expected response cannot be deduced from the input signal. According to Vossius (1980), there is also limited observability and controllability. That is, an arbitrary initial state cannot be reconstructed from the system response, nor can it be transferred to an arbitrary final state [5]. Therapeutically, this means that it is never possible to reach conclusions about the initial state before the symptoms [5]. This leads to various forms of fuzziness: (a) the uncertainty of a countercorrection to the input interference, (b) the decentralization of corrections in highly complex systems, (c) the imprecision of information flow, and (d) the uncertainty regarding the lack of a direct cause.

Only the renunciation of the determining of details (fuzziness) allows for the control of a higher degree of complexity. In this context, fuzziness is not necessarily negative. It is the counterpart to the pure accumulation of data. For example, fuzziness also plays a role in pattern recognition in medicine, such as in manual therapy [41]. Fuzziness as a basis for pattern recognition leads to the following conclusions for the therapy of palliative patients: (1) common goals instead of patriarchal therapy hierarchy, (2) establishing resources for finding identity, i.e., what else can the patient do? and (3) not treating the disruptive factors (such as pain) but making it a priority to make the system less sensitive to the disruptive factors/pain [41].

Biopsychosocial model: From the perspective of palliative care, fuzziness also means that it is not the pure science with all its exact data alone that are important but also the “soft data” [30], for example, the psychological state and the social context [29] without attributing causality. In addition, the psychosocial aspect is emphasized together with the biological-medical aspect [58]. Additionally, the psychosocial aspect is emphasized along with the biological-medical aspect. According to Mounsey et al. (2018), recognizing the importance of psychosocial factors means realising “psychosocial care”, “support for families”, and others [60]. According to Latimer and Dawson (1993), biopsychosocial care indicates “physical and emotional care” [59]. The biopsychosocial model of palliative care was expanded by Cicely Saunders to include the spiritual factor [70]. However, even she did not solve the main problem of the biopsychosocial model. According to Engel (1977), the biopsychosocial model is often exhausted in the description of the individual components bio, psycho, and social [71,72,73,74,75], without clarifying the relationship between the individual factors. The psyche from the point of view of somatic medicine is evaluated as reactive to somatic disorders [26]. This approach is not sufficiently different from the mechanical model, according to Descartes (1969) [76,77,78]. The psychosocial alone is more: it refers to the totality of all environmental conditions before [79], during, and after the onset of symptoms [80]. The biopsychosocial model has therefore been criticized for several years [76,77,81]. The same applies to the concept of total pain.

From the perspective of cybernetics, cybernetic models include biological, psychological, and social systems, in contrast to mechanical models in which psychological and social processes do not play a role. Thus, cybernetic systems are always linked to the environment [5]. In his remarks on applied cybernetics in medicine, Vester (2015) concludes that no matter how much technical effort is expended [30], it does not lead anywhere if one passes by the core of maintaining health. This core is the harmonious interaction of the manifold biological processes in an organism and its interactions with its environment and fellow human beings. This is also valid for diseases because, according to Vester, diseases can occur too fast to treat the disturbance; thus, a partial goal is aimed at without considering the associated changes in the total structure (see also point 1, quality of life).

Dualism means constant exchange with the environment. Learning by adaptation take place in these systems by considering the conditions for changes at one level. It is not possible to keep the initial conditions of a biological system constant because they are constantly changing. The succession of changes is the so-called “connectivity of the system” [82], that is, the ability of a system to reproduce repeatedly.

Since these are biological systems, there is no thermodynamic equilibrium. This imbalance triggers self-organizing processes and thereby maintains metabolism [36]. This system, presenting as an energetically open (and operationally closed) system, undergoes changes, but only within a given framework determined by genetics [37]. This is the basis of the identity of the subject in transition. For transition, this means recognizing that an individual is autonomous but not self-sufficient. Transition also means that the individual as a social being is also defined by how he or she deals with the biological, psychological, and social influences from the environment.

Social isolation then leads to the collapse of the system [69].

Therapy vs. symptom control: From the perspective of palliative care, therapy in the true sense is the elimination of symptoms. In contrast, palliative care is about symptom control. This means optimizing the patient’s condition while monitoring the symptom [83]. Prior to starting therapy, an appropriate prioritization of the identified components ascertained (reaction or cause or comorbidity) must take place [84,85]. If this is no longer possible due to the disease, a new equilibrium must be created for the patient on the basis of quality of life and satisfaction [86]. By establishing new resources, the individual can be given a new identity. For therapy, this means that the goal is not solely to alleviate suffering but to create new resources for compensation [87]. Improving resources means, for example, improving social competence through psychotherapy or treating phobias/anxiety disorders with psychotropic drugs [88].

From the perspective of cybernetics, the starting point and feedback are worth mentioning here. Regarding the starting point, biological systems are open to external stimuli. Therefore, it is not possible to keep the initial conditions of a biological system constant [6], since it is constantly changing (through adaptation). Symptoms are already the endpoint of a process [5]. Because of the constant change, there is also no returning to the starting point [82]: “Everything that happens, happens for the first and for the last time”). Regarding feedback, self-organizing biological systems do not correct themselves back to the initial data before the disturbance [4,5] but to a new equilibrium (which never occurs in place completely, see above). From a cybernetic perspective, biopsychosocial means a permissive understanding of the development of chronic symptoms [85]. This means that the principle is not constant repetition but a learning process through adaptation to an unexpected disruptive factor [89].

Chronification processes, such as chronic pain, do not develop as a pure learning process but through the failure of the negative feedback in the cycle [5]. The symptomatology arises at the moment when the reversal of signs after the actuator turns into positive feedback, meaning that the disturbance factor, or its direct effects, are amplified instead of counter-regulated [90]. This is the so-called tipping point. The disturbance factor pain as an external stimulus (that caused the feedback) is not part of this cybernetic cycle [5]. Thus, the disturbance factor is not a reducible quantity in system-theoretical models and is therefore not self-corrected [25] but only counter-regulated. Therefore, the counter-regulation is not carried out as restitutio ad integrum [5], i.e., to the starting point. Moreover, the disturbance factor is not to be eliminated; the system is to be made insensitive to the disturbance factor and fault-tolerant [30,41]. Thus, being symptom-free (pain, thirst, constipation) is not a control variable [30] but a state of the system [69].

Regarding resources in cybernetics, the outcome of the process also requires the consideration of resources and the possible sustaining factors [91] with the ability to counteract [79,92]. The assumption of an operationally closed self-organizing system also makes the resources/buffers necessary [30].

Regarding controllers in cybernetics, and the property of defining only abstract control objectives, the correction of a disturbance factor does not require a supreme control authority [30]. A controller in cybernetic systems has a target value. This target value is a variable assessment of pain or pain-related impairment, for example. It is subject to various influences, such as the specifications of the body [5]. Thus, an improvement in the factors influencing the target value can lead to a more favorable evaluation of pain. Resistance to therapy might also be due to a change in the target value.

Regarding the speed of therapy in cybernetics, there is no quick “cause-and-consequence” principle [5]. Thus, for example, in pain therapy, in contrast to acute therapy, success can only be achieved at a slow pace in cybernetic understanding because the target value is readjusted repeatedly [5].

Cybernetic systems can also be pushed to their limits, such as diseases in the measured value controller area or in the actuators [5]. The same applies to a massive, too fast adjustment of the target value. A very large disturbance can lead to over-control of the system: the regulator makes corrections until the system is exhausted. This leads to a decrease in the controlled system below the setpoint value until the system can resume counter-regulation. As usual, the system does not adjust to the setpoint value and fails to stabilize, which can ultimately lead to the destruction of the system [69,93]. This implies that massive complaints should not lead to a massive therapeutic counter-reactions [69].

Autonomy of the patient: From the perspective of palliative care, the autonomy of the patient means the perception of one’s own body as “normal”, combined with the desire to be able to continue to perform the activities of daily living [20] and continue to maintain control over one’s own life. Autonomy requires being treated with respect and with mutual trust and being treated as an individual [20]. Latimer and Dawson (1993) formulated the following principles of palliative care: the patient must be allowed to live the remainder of his or her life in accordance with his or her previous belief system, personality, and values [59]. Whenever possible, the care system must be flexible: (a) easy and early access to services as the needs dictate, and (b) a variety of settings for care are needed. The patient’s autonomy also extends to the termination of life at the end of life and the ethical issues of suicide [58,61]. Autonomy is also the basis of the living will. Reindl (2012) equates autonomy with autopoiesis [94]. This is also the view of Hochwarter (2011) [95]. Reindl (2012) draws the following conclusions: (1) To claim autonomy, the system must be closed. That is, it is the patient who decides, not the doctor, not the politician, etc. (2) Decisions are made according to the patient’s own will. This does not mean the arbitrariness pushed by the critics, which would be in line with a demand for autarky. (3) Every system also needs an environment from which it differs; otherwise, it is not a system. This is where the doctor comes in. (4) The patient is responsible for himself and his decisions, but (5) he needs appropriate input from the outside for this—because autonomy means neither complete independence nor arbitrariness in the sense claimed by the critics. (6) For this to happen, the patient must be enabled to see himself as a closed system in the first place [94]. This is again a return to the first step and shows recursivity.

In addition, because of the many distortions in the system, only part of the information received from the patient is likely to be truly relevant to his or her concerns. Nevertheless, dealing with such a patient means more than just eliminating symptoms [96,97,98].

From a cybernetic perspective, the concept of autonomy has two roots: autopoiesis [94] and the unique position of the human being [47]. According to Maturana (quoted by [36]), autopoiesis is the mechanism that makes living beings autonomous systems.

In applied cybernetics, according to Wolf (1990), self-organization also requires self-discipline [31]. Instead of creating a monopoly on information, it is helpful to communicate with the patient based on agreed rules. An action/attitude is not better a priori. Only compliance with the rules of the game is correct [31].

#### 4.2.2. Cybernetic Terms without Corresponding Palliative Care Equivalents

In palliative care, quality of life, treatment of disturbance variables/negative feedback (symptom control), and dualism have equivalents in cybernetics. Some other terms (complexity, stability, rhythmics) do not have equivalents (thus far) but are discussed in diploma theses/dissertations on palliative care or publications on tumor therapy.

Complexity vs. hierarchy: From the perspective of palliative care, the main problem with the use of the term complexity is that in German, complex is confused with complicated [30,99]. Therefore, a cybernetic understanding of complexity in the sense of the mutual relations of systems is conceivable as a basis for palliative care action if palliative care sees as a goal the integration of the psychological, social, and pastoral needs of the patient, the relatives, and the treatment team both during the illness and while dying [58]. As shown in Figure 3, in palliative care, too, a purely hierarchical structure is only possible up to a certain point: when the complexity of an organization exceeds the control or capacity of a hierarchy [100]. A higher efficacy results from self-organization. However, networking alone does not result in stability (Figure 3). Complex systems become more stable through the formation of subunits. In the context of palliative care, this would be the theoretical background for working in small teams.

From the perspective of cybernetics, systems are complex when their parts are interconnected by mutual, permanently changing relationships [101]. The error of linear–causal thinking discussed at the beginning of this text also has a consequence: the assumption of hierarchy. According to Weiner (1994), this does not have to be because the case because, for example, mental processes do not need command cells [6]. As shown in Figure 3, cybernetics presents a departure from hierarchical structures. Complex cybernetic systems with their networked self-organization come into consideration above all when the complexity of an organization exceeds the control capacity of a hierarchy [100]. Cybernetic systems in highly complex structures thus depend on self-organization. Since the hierarchy is missing, the origin of a considered level is undefined. A constant increase in complexity does not exist in self-organized systems.

Stability: From a palliative care perspective, stability involves identity and decay. In palliative care, one’s identity with oneself is related to the idea of autonomy as formulated by Houska and Loučka (2019): being “normal” for as long as possible and being able to perform the usual daily activities while considering retaining dignity [20]. The authors have concretized both in further points, especially the point of dignity, which was elaborated among others in meaning “to maintain dignity and integrity”. Palliative care is active and compassionate care primarily directed toward improving the quality of life of people who are dying and supporting patients and families as they experience multiple losses [59]. Mounsey et al. (2018) states that palliative care is often linked to the care of people with cancer, for whom symptom burdens and illness trajectories have been largely predictable, with the time of functional preservation being followed by a rapid terminal decline [60].

Hochwarter (2011) writes in her diploma thesis on palliative care in children that structures are by no means static [95]. They are only temporarily stable. They can be modified because they are produced in the moment of autopoietic (re)production. Therefore, they are valid only for as long as the evolutionary process persists and exchanges them against another structure. Evolution is only possible because everything the system consists of must be continually renewed. The factor time is therefore constitutive for autopoiesis. We did not find any good definition of the term “identity” in the literature on palliative care (outside the framework of cultural sensitivity). Perhaps the best way to approach the term is through the psychiatric psychopathology concept of “I” [102]. What does it mean, if we say, I walk, I decide?

1. Activity. Actions are provided by me.
2. Unity of the “I”: I can never break down into several independent ego parts.
3. Continuity of “I”: Yesterday I was me. Today I am, too.
4. Independence of the “I”: I am no one other than myself.

Previously unanswered questions in palliative care arise from these questions: if there is no stability at the end of life, does the identity change as well? Does the patient’s identity change with decay?

Stability, identity, anticipation, and error of cybernetic self-organizing living systems are next discussed from the perspective of cybernetics. The second feature of cybernetic self-organizing living systems is stability. Networking instead of hierarchy creates stability. However, networking is also limited. At a certain degree of networking, the stability of the system is possible only if it forms subunits that are connected to each other only by the specifications of the necessities of the system [30]. (As described above for palliative care.) This also includes the instability of biological systems [95] in terms of a permanent change, according to Etxeberria (2004) [37]. Niedermüller and Hofecker (1990) state that by keeping self-organizing processes going, the dynamics of a structure that never rests would emerge [36].

Biological systems imply a continuous change simply because of the impossibility of a thermodynamic equilibrium (which is equivalent to death). When a system undergoes changes, it is alive. Thus, change (which is further determined by the genome [37] is to be regarded as a characteristic of stability. However, in cybernetics, continuity is not created by a succession of individual operations but rather by the perpetual synchronization of the system of goals and results [82,103,104]. Stability is guaranteed only by that level of self-organization at which the order can tolerate disruptions without the need to give up its own values [31].

Ultimately, it is about the paradox of constant change on the one hand and identity on the other. Identity is created by the rules within which transformation takes place [82]. Otherwise, the system, as an energetically open (and operationally closed) system, changes only within a given framework determined by genetics (Etxeberria, 2004) [37]. Von Uexküll and Wesiak (1991) solved the problem of the identity of systems that constantly change their permanence and their structure by stating that this paradox is an essential feature of all living entities [4].

Regarding anticipation, cybernetic systems do not only react with the environment at a certain point in time. According to Rosen (1978), the existence of preformed systems also determines the ability to respond to future demands on the system (ability of anticipation) [105]. The more precisely the system is programmed to cope with future changes, the longer the lifespan of the system [36]. Unfortunately, there may be discrepancies between the prediction and actual change, which may result in an increasing incompetence of positive feedback processes (feedforwards), thus limiting the functional lifetime of the system [106].

Because of the preformed rules, self-replication introduces errors that are not conducive to identity. This is the case when the complexity of the system increases [106]. According to Niedermüller and Hofecker (1990), continuous change is always accompanied by errors [36]. This is due, among other things, to renewal, which will never be fully completed. As a result, errors, such as an accumulation of less active enzymes in old age, can occur. The same conditions that made autopoiesis possibility (energetic openness, inequality, defectiveness of information transmission) also imply the possibility of internal self-reinforcement of defective fluctuations [36], which eventually cannot be corrected by the system as a whole. This means that the lifespan is shortened, or oncological processes develop.

Rhythmics: From a palliative care perspective, according to Sittl (1995), cancer pain in 70% of cancer patients with neuropathic pain shows rhythmicity in pain sensation, and 40% of these patients report a maximum pain in the early evening hours [106]. The condition of a patient is to be determined by more than only a momentary observation. Due to the oscillations of many processes of the human being, such as the psyche (e.g., in cyclothymia or hypertension), catamnesis (a longitudinal observation) is the only appropriate approach and may help the patient [5].

From the perspective of cybernetics, rhythmics is considered the third property of cybernetic-self-organizing (autopoietic) living systems. According to Weiner (1994), fluctuations and rhythmic oscillations are characteristic of numerous natural phenomena, such as the diurnal (physiological) fluctuations of the parasympathetic nervous system and the seasonal (nonphysiological) fluctuations of depression [6]. For example, various parameters of heart rate variability from the frequency domain can be assigned to physiological processes (fluctuations in thermoregulation, oscillations of arterial blood pressure, respiratory oscillations/respiratory sinus arrhythmia) [7].

Time behavior: The difference between the delayed time sequences and the environment, which works in real time, is significant [1]; this difference can be the starting point for the development of diseases. While temporal behavior plays a role in cybernetics, this term has not yet arrived in palliative care.

### 4.3. The Role of Psychophysiology in Palliative Care

The intersection of cybernetics and psychophysiology is the subjectivity of the individual [1]. Psychophysiology is based on three principles [1]:

1. Physiological processes are measurable (EEG, 24-h ECG);
2. They are not identical in all people, but are individual;
3. There is no compelling relationship between observable/observable physiological parameters and the conditions perceived by the patient.

The common basis of these principles is the subjectivity of the individual, which is found in psychophysiology and is important for palliative care. It has several aspects:

- Interoception is the perception of one’s own psychophysical states.
- Subjective perception was also identified by Brinkers as a key factor in the assessment of the overall clinical picture [15] (see also [1]).
- Personality traits.

Brinkers recently pointed out the importance of disposition for the development of physical and/or psychological complaints. In psychiatry, this corresponds to the diathesis–stress model [15].

Tipping Point/Overload

According to the Meikirch model, illness is a mismatch between the individual’s potential and the demands placed on it. In psychophysiology, this corresponds to the overloading of the system [21] in cybernetics to the tipping point at which the counter-regulation of a disruptive factor becomes positive reinforcement.

What psychophysiology also has in common with cybernetics is that it is interested in the borderline area from the “normal state” to the incipient state of illness, to the development of clinical symptoms. In cybernetics, this is the tipping point at which negative feedback/counter-regulation turns into positive reinforcement. In psychophysiology, this is the overload of a system [14].

Palliative care no longer seems to be about the symptom development. However, this is misleading, as palliative care patients can also develop pain and anxiety after an incurable illness has occurred. The present review focuses on the topics of consciousness, anxiety, and chronic pain, which are of interest in psychophysiology [2,14], but also in palliative care [11,44,45,46].

In addition, subjectivity plays a much greater role in palliative care, as it is no longer about healing, but about optimizing the personal psychophysiological situation. However, the question arises as to how long subjectivity is important in the dying process. Palliative care distinguishes four phases at the end of a life marked by an incurable illness [107].

(a) A preliminary phase or rehabilitation phase:

In this phase, the patient can be largely reintegrated into normal social life through palliative therapy despite the advanced stage of the illness.

(b) Preterminal phase:

The patient shows clearly visible symptoms of the advanced disease. Nevertheless, most symptoms such as pain can be alleviated through symptom control. The prognosis at this stage is several weeks to months.

(c) Terminal phase:

The seriously ill patient is living on the verge of death. The patient withdraws his attention more and more from the environment and says goodbye. The prognosis consists of a few days to a week.

(d) Final phase:

The person is dying. The actual onset of death is foreseeable. The time course of the dying process is variable within limits.

The fields of cybernetics and psychophysiology have in common the system–environment dualism, the counter-regulation in the circulatory model, subjectivity in the form of personal environmental conditions, and subjective perception as well as personality traits/diathesis. Measures such as biofeedback [24,51] are based on these. Cybernetics and psychophysiology also have parameters such as time shift and rhythm that have not yet been discussed in palliative care (see above).

Important for palliative care and common to psychophysiology are the disorders of consciousness, anxiety, and pain, which are not part of cybernetics. In palliative care, they are treated according to the cybernetic principles of symptom control rather than causal therapy, the quality of life of the individual, constant adaptation to the psychosocial environment, and respect for the autonomy and dignity of the individual. The therapies culminate in the one principle that in palliative care everything can be performed that is good for the individual.

It is important to note that some of the therapeutic measures developed on the basis of the cybernetic principles of psychophysiology (biofeedback, hypnosis, music, aromatherapy) can also be applied to the cybernetic principles of palliative care and are helpful [3,10,11,24,44,51]. This means the theoretical target is strengthening the terminally ill person in the context of bio-psycho-social influences and making him or her less sensitive to disturbances/dysregulation. From the perspective of psychophysiology, it refers to the positive influence of whatever feels good for the patients in their limited psychophysical state.

#### 4.3.1. Symptom Burden (of Anxiety and Pain) in General

Symptom burden itself is a topic of both psychophysiology and palliative care. According to Koesel et al., the symptom burden itself (burden of anxiety and pain) can be improved simply by observing guidelines for patients [108].

#### 4.3.2. Anxiety and Pain

Music therapy: In his review of the effect of music on the body, mind, and cognition, Trappe also looked at the effect of music on pain therapy for the dying [10]. He referred to studies that have shown that music in intensive care units can often make high-dose treatments with analgesics or sedatives superfluous. In addition, the sensation of pain is greatly reduced. The body’s own hormones are released, which have a pain-relieving and mood-enhancing effect. Trappe argues that a dying person is often “still hearing”, even in the last phase of life, when many senses and organs have otherwise already shut down.

For this reason, according to Mramor, music is becoming increasingly important, especially for the dying and in hospices [109].

Massage: According to Falkensteiner et al., massage reduces the subjectively perceived pain symptom in oncology patients as well as anxiety [110]. However, according to Jane et al. [110,111], this effect of relaxation only lasts a few hours after the end of the massage.

Biofeedback: Against a cybernetic background, it can be concluded that biofeedback provides a new feedback loop that can help individuals regain physiological self-control [24]. According to Tsai 2007 et al., the pain-relieving effect of biofeedback is achieved through muscle relaxation [51]. According to the authors, the underlying mechanism is attenuation of physiological arousal. At the same time, however, the authors also stated that this does not work if the tumor patients have massive psychological disorders; in this case, they recommended a combination with psychotropic drugs.

#### 4.3.3. Consciousness

Parts of the definition of consciousness are also the subject of psychophysiological research (e.g., perception, attention [2]). The objective, reliable, and valid measurement of the state of consciousness is still a problem [46]. Nevertheless, there are therapy methods that have an effect on consciousness in palliative care patients.

Aromatherapy: In a study of 15 conscious patients and 5 unconscious palliative care patients compared with healthy controls, Goepfert et al. used these two essences (lemon oil and lavender) [11]. Respiratory rate, heart rate, oxygen saturation, blood pressure, and mean arterial pressure were measured. They came to the following results:

Healthy volunteers responded to lemon oil with a significant increase in respiratory rate, heart rate, and diastolic blood pressure, while a significant decrease in respiratory rate was measured in response to lavender oil. Conscious and unconscious palliative patients responded to lemon oil with a significant increase in all measured parameters and to lavender oil with a significant decrease in all parameters except oxygen saturation. Conclusion: healthy patients show less response reactions than palliative patients—regardless of their state of consciousness.

Hypnosis: Many publications deal with the effect of hypnosis on pain and anxiety. Bissonnette et al. found a positive effect of hypnosis in combination with music in a meta-analysis in 2022 [3,10], but did not state a presumed basis for the effect. Facco et al. consider the therapeutic goal (and mode of action?) of hypnosis to be that the palliative patient regains control over body and mind (symptom control) [44]. Brugnoli et al. point out that hypnosis serves to modify physical function as well as behavior via the mind and thus also to optimize psychological (anxiety) and physical (pain) factors [52].

### 4.4. Strengths, Limitations, and Criticism of the Narrative Review

#### 4.4.1. Strengths

This paper presents the first investigation of cybernetic and psychophysiological terms in palliative care. So far there is no knowledge about this.

It has now been possible to show which palliative care terms have a clinical basis. For some palliative care terms, cybernetic thinking is already present (quality of life, autonomy, symptom control), while for others it is not (biopsychosocial). Cybernetic terms (complexity, stability, identity, rhythm) are still used to a lesser extent in palliative care. Other terms associated with palliative care (e.g., communication, human vs. machine) and their equivalents in other fields could be discussed.

Based on the comparisons of cybernetic terms and psychophysiology and their relevance to palliative care, therapies based on these two fields can then be applied to palliative care on a scientific basis: awareness therapy, aromatherapy, music therapy.

#### 4.4.2. Limitations

As the first study of its kind in palliative care, it does not claim to be exhaustive. It would have been desirable if some studies on the topic used here had already been available.

#### 4.4.3. Criticism

Schmidgen has already discussed the subjectivity of perception in the context of cybernetics. This aspect was also considered here. This made the overview very long. Future work can be devoted to individual aspects.

## 5. Conclusions

This review examined the extent to which cybernetic and psychophysiological thinking is present in psychophysiology and palliative care. The following key statements can be made.

1. The common theme of cybernetic thinking, psychophysiology, and palliative care is the concept of subjectivity.
2. Cybernetics and psychophysiology are significant for palliative care within the concepts of quality of life, autonomy, and symptom control.
3. Works on applied cybernetics in biology, psychophysiology, and business management demonstrate the practical applicability of the notions of complexity, identity, rhythmicity, and temporal behavior. However, these have not yet been formally used in palliative care.
4. Concrete measures and therapies for the subjective benefit of palliative patients are based in particular on psychophysiology (bio-feedback, hypnosis).
5. Cybernetics teaches that networked systems are superior to hierarchical structures in solving problems. This is a consequence of pattern recognition rather than data accumulation and the rejection of local solutions. For palliative care, the comparison with cybernetic thinking means that therapy is not a matter of exhausting the maximum or restitutio ad integrum, but of “going with the flow” in recognition of what is still possible for the patient, regardless of what has determined the sense of the limits of life. This is a central demand not only of psychophysiology, but of palliative care in general.
6. The assumption of a cybernetic biopsychosocial system would pay more attention to the remaining possibilities, dispositions, and subjective requirements of the individual. All of this is the basis of psychophysiology in particular.
7. It would be advisable for palliative care to take a closer look at these issues.

Outlook into the future: The review shows the following:

Cybernetic thinking is already present in some palliative care terms (quality of life, autonomy, symptom control), but not in others (biopsychosocial). Cybernetic terms such as complexity, stability, identity, temporal behavior of systems, and rhythm are still less used in palliative care. Palliative care would benefit from working out the meaning and consequences of these issues for itself.

## Figures and Tables

**Table 1:** Comparison of physical and biological systems (Weiner, 1994 [6]).

	Physical	Biological
Formal	Closed system	Open system
Balance	Stationary	Flowing/dynamic
Operation	Exact	Fuzzy
Final state	Heat death	Cold death [4,6]
Correction	To original state, “hostile to history” [4]	New equilibrium

## Data Availability

Not applicable.
